# Crosstalk between Resveratrol and Gut Barrier: A Review

**DOI:** 10.3390/ijms232315279

**Published:** 2022-12-03

**Authors:** Natalia Drabińska, Elżbieta Jarocka-Cyrta

**Affiliations:** 1 Department of Chemistry and Biodynamics of Food, Institute of Animal Reproduction and Food Research of Polish Academy of Sciences, 10-748 Olsztyn, Poland; 2 Department of Pediatrics, Gastroenterology and Nutrition, Faculty of Medicine, Collegium Medicum, University of Warmia and Mazury, Żołnierska 18A Str., 10-561 Olsztyn, Poland

**Keywords:** intestinal permeability, gut barrier, leaky gut, polyphenols, resveratrol

## Abstract

The plant-based nutraceuticals are receiving increasing interest in recent time. The high attraction to the phytochemicals is associated with their anti-inflammatory and antioxidant activities, which can lead to reduced risk of the development of cardiovascular and other non-communicable diseases. One of the most disseminated groups of plant bioactives are phenolic compounds. It was recently hypothesized that phenolic compounds can have the ability to improve the functioning of the gut barrier. The available studies showed that one of the polyphenols, resveratrol, has great potential to improve the integrity of the gut barrier. Very promising results have been obtained with in vitro and animal models. Still, more clinical trials must be performed to evaluate the effect of resveratrol on the gut barrier, especially in individuals with increased intestinal permeability. Moreover, the interplay between phenolic compounds, intestinal microbiota and gut barrier should be carefully evaluated in the future. Therefore, this review offers an overview of the current knowledge about the interaction between polyphenols with a special emphasis on resveratrol and the gut barrier, summarizes the available methods to evaluate the intestinal permeability, discusses the current research gaps and proposes the directions for future studies in this research area.

## 1. Introduction

**Figure 1:**
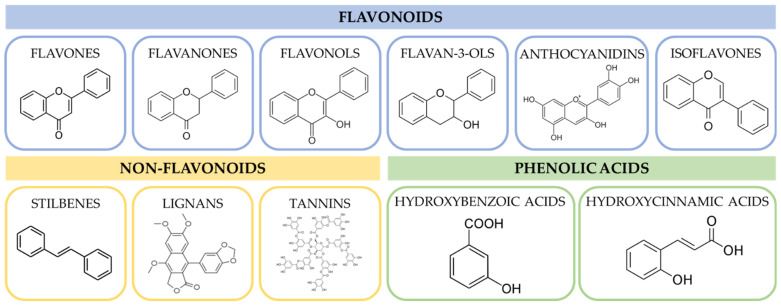
The classification of phenolic compounds.

The interest in plant-based nutraceuticals is growing over the last decades. Many of the phytochemicals were found to exhibit anti-inflammatory and antioxidant effects, which can reduce the risk of the development of cardiovascular and other non-communicable diseases. One of the most disseminated groups of plant bioactives are phenolic compounds. Phenolic compounds are a very diverse group of secondary plant metabolites, which are widely distributed in vegetables, fruits, nuts and various plant-based food. The biological function of these compounds in plants is generally involved in protection against pathogens and herbivores, the attraction of pollinators, and the defence against ultraviolet radiation [1]. Phenolic compounds are formed from acetyl coenzyme A and amino acids as precursors or via the shikimic acid pathway. In the latter case, the carbohydrate precursors of the glycolysis and pentose phosphate pathway are converted into tyrosine, phenylalanine and tryptophan, which are then deaminated enzymatically to form cinnamic acid [1]. The group of phenolic compounds consists of approx. 8000 compounds, which can be further categorised into classes as presented in Figure 1. The simplest phenolic compound is phenol, which is a primary structural unit in phenolic compounds. The flavonoids consist of two benzene rings bound with a heterogenous pyrone C ring, and the subclasses of flavonoids differ with the number and the position of hydroxyl groups, which determine their biological functions [2]. The non-flavonoids consist of the simplest benzoic acid to very complex structures such as lignans and tannins. The main health-beneficial functions of phenolic compounds are associated with their anti-inflammatory and antioxidant properties due to the ability of phenolic compounds to donate hydrogen or electrons to free radicals. Consequently, it leads to the stabilization of cell membranes, protects from cellular oxidative stress and limits the production of pro-inflammatory cytokines such as interleukin (IL) 6 and 8 as well as tumour necrosis factor α (TNF-α) [3,4]. The bioactive activity of polyphenols has been extensively studied and summarized in excellent reviews [5,6,7].

Ingested phenolic compounds must be absorbed in the intestinal tract to exhibit their systemic effects. The absorption of phenolic compounds depends on the molecular weight, stereochemistry, presence of specific functional groups and lipophilicity [8]. Some of the phenolic compounds are present in free forms. However, generally, they are bound to proteins or sugars. Small molecular weight phenolic compounds, such as isoflavones and gallic acid, are easily absorbed into the intestinal epithelium [9]. Contrary, many of the phenolic compounds are absorbed only in 0.3–43%, therefore their concentration in the circulation is low [9]. It is hypothesized that phenolic compounds are transported by passive diffusion or by certain transporters such as sodium-dependent glucose transporter 1 (SGLT1), glucose transporter 2 (GLUT2) and P-glycoprotein, which are present in the cell membranes [10,11].

The metabolism and degradation of some phenolic compounds, such as anthocyanins, occur partly already in the oral cavity, which is moderately mediated by local microbiota [12]. Other compounds are hydrolysed in the stomach, and small intestine (5–10%), while many phenolic compounds reach the colon in the intact form [13]. The absorption of phenolic compounds in the duodenum and jejunum is closely related to the activity of digestive enzymes, which release aglycons from the food matrix. The enzymes involved in the transformation of the phenolic compound include among others lactase-phlorizin hydrolase and cytosolic β-glucosidase [14,15]. The majority of phenolic compounds is transported to the colon, where they are subjected to the enzymatic activity of gut microbiota. The intestinal bacteria degrade the aromatic ring and release aglycones facilitating the absorption of phenolic compound derivatives. Generally, the polymeric phenolics are hydrolysed to phenolic acids, flavones and flavanones to hydroxyphenylpropionic acid, flavanols to hydroxyphenyl acetic acid and proanthocyanidins to phenolic acids of a smaller molecular weight, which then can be absorbed [15].

A new branch of studies focuses on the interaction between polyphenols and gut microbiota. The mechanisms of these interactions are not fully understood. However, it is suggested that polyphenols have stimulating activity on the gut bacteria, and therefore according to the new classification of the International Scientific Association for Probiotics and Prebiotics (ISAPP) are classified as prebiotics [16]. Moreover, some studies reported that the simultaneous intake of polyphenols with probiotics can enhance the positive effects of the latter [2]. On the other hand, gut bacteria are suggested to be involved in the metabolism of phenolic compounds, which can lead to their better bioavailability [17]. Recently, it is hypothesized that polyphenols can be potential modulators of intestinal permeability, although the mechanism is not fully understood. Therefore, this review offers an overview of the current knowledge about the interaction between polyphenols with a special emphasis on resveratrol and the gut barrier, discusses the current research gaps and proposes the directions for future studies in this research area. Moreover, the available methods used for the evaluation of the gut barrier integrity are summarized and their limitations are discussed.

## 2. Resveratrol—Overview

**Figure 2:**
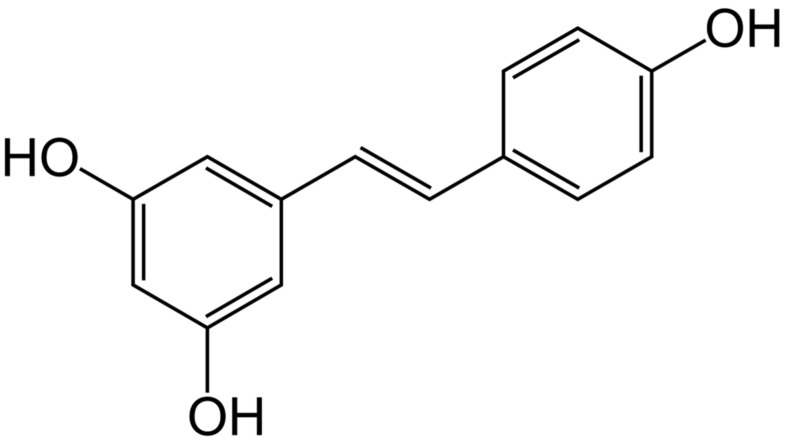
The chemical structure of resveratrol.

Resveratrol (3,5,4′-trihydroxystilbene) belongs to the group of stilbenes, which are characterized by two phenol rings linked by an ethylene bridge, and its structure is presented in Figure 2. Resveratrol can exist in two forms: *cis* and *trans*, and the latter is more prevalent and potent than the other. The interest in resveratrol is associated with its high antioxidant potential. Many studies have reported that resveratrol possesses anti-cancerogenic, anti-inflammatory and neuroprotective activity [18,19]. Resveratrol can be detected in over 70 plant species. However, the highest content is detected in grapes, peanuts and berries [19]. Early studies showed that high content of resveratrol was present in injured and infected plants. Currently it is known that stilbenes, in general, provide protection against microbial and fungal infections in plants and therefore they are classified as phytoalexins [20]. In plants, resveratrol is present in the glycosylated form to protect it from enzymatic oxidation and hence increasing its stability and consequently preserving the biological effects [19]. Resveratrol is suggested to be responsible for the “French paradox” defined by Renauld and Lorgeril in 1992 [21], who noticed a lower rate of heart diseases in Southern French who regularly drink red wine, despite their diet being rich in saturated fat.

Resveratrol has the ability to activate sirtuin 1 (SIRT1), which deacetylates histones and nonhistone proteins, including transcription factors [22]. SIRT1 is involved in various metabolic processes, including stress resistance, cellular senescence, endothelial functions, and cell survival, thus it is suggested that resveratrol can be beneficial in diseases associated with inflammation, cell cycle defects and metabolic disturbances [18]. Moreover, resveratrol was found to influence the signalling pathways of the nuclear factor κβ (NF-κβ), insulin-like growth factor type 1 receptor (IGF-1R)/Wnt, the mechanistic target of rapamycin complex 1 (mTORC1), Akt/mTORC1/S6K1 and others [23,24,25,26], which are involved in carcinogenesis and the development of cardiovascular, metabolic and neurodegenerative diseases.

When discussing the health-beneficial potential of plant bioactive compounds, the concepts of bioaccessibility, bioavailability and bioactivity have to be defined. Bioaccessibility is defined as the quantity which is released from the food matrix in the intestinal tract and becomes available for absorption. Next, bioavailability can be defined as the fraction of a compound and/or its bioactive metabolites which reach systemic circulation. Finally, bioactivity includes the specific physiological response to the presence of a certain substance [9]. The bioavailability of resveratrol was established as relatively low and this polyphenol is rapidly metabolised after oral ingestion [27]. Approx. 80% of resveratrol is absorbed in the intestine and the free form is bound to albumins and lipoproteins, which serve as a reservoir and distributor of resveratrol [28]. However, after absorption, resveratrol is metabolized by the liver, forming two forms of resveratrol, glucuronidated and sulphated, which can have lower beneficial activity compared to resveratrol itself [28]. Collectively, it is considered that from 80% absorbed, only approx. 1% of free resveratrol is bioavailable [27]. Furthermore, the absorbed resveratrol is rapidly excreted in approx. 75% in urine and cannot be utilized by the organism. Therefore, many of the results conducted in vitro where the substance is directly placed on the cells did not have the confirmation by in vivo studies applying the same doses, which results from the metabolic changes of the compound.

The study with isotopically-labelled resveratrol showed that next to glucuronidated and sulphated forms of resveratrol, a third conjugated form resulting from microbial activity is detected in human urine [29]. All three metabolites accounted for 25% of resveratrol ingested. It can suggest that the intestinal microbiota plays an important role in the bioavailability of resveratrol and its biological functions. Later studies discovered that resveratrol reaching the colon is converted by gut bacteria mainly to dihydroresveratrol (3,4-dihydroxystilbene) and lunularin (3,4′-dihydroxybibenzyl) [30]. The bacteria responsible for this conversion were identified as *Slackia equolifaciens* and *Adlercreutzia equolifaciens* [30]. It is hypothesized that the conjugated forms of resveratrol, especially the glucuronidated ones, serve as reservoirs of this compound from which it can be released locally by tissue β-glucuronidases. However, it was not confirmed experimentally yet [27]. In addition, it has to be kept in mind that also gut bacteria can produce this enzyme, increasing resveratrol bioavailability [31].

## 3. Gut Barrier—Structure and Importance

**Figure 3:**
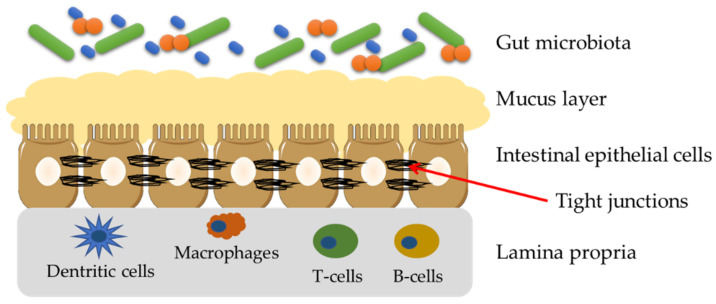
The scheme of the gut barrier.

A gut barrier is a functional unit organized into intestinal microbiota, a mucus layer, intestinal epithelial cells (IECs) and lamina propria (Figure 3). A physical barrier consists of IECs, which are sealed by tight junctions (TJs), adherens junctions (AJs) and desmosomes [32]. TJs which are found on the side parts of enterocytes are built of transmembrane proteins such as claudins, occludins, peripheral membrane proteins, i.e., zonula occludens (ZO), and regulatory proteins. The most flexible part of the gut barrier is microbiota, which composition and count depend on lifestyle, diet and ongoing diseases. The microbiota plays an important role in the gut barrier integrity and function by adherence of commensal bacteria to the intestinal mucosa and forming an additional, protective layer [33,34].

The functioning of the intestinal barrier is a dynamic process, dependent on the activity of intercellular connections, regulated by both dietary components, the nervous system, inflammatory mediators and hormones. The intestinal barrier is, therefore, responsible for maintaining the balance between the selective permeability of nutrients from the intestinal lumen into the circulation and the internal milieu as well as the protection of the organism against the penetration of harmful components of the external environment. Under physiological conditions, the selective absorption of nutrients through intercellular transport occurs, while unnecessary food components and harmful substances are removed from the gastrointestinal tract. The disruption of the proper functioning of the intestinal barrier by violating its integrity may result in the development of inflammation, as a consequence of uncontrolled penetration of antigens and products of bacterial metabolism. The impairment of the gut barrier has been associated with the development of various diseases, including celiac disease, obesity, non-alcoholic steatohepatitis (NASH) or non-alcoholic fatty liver disease (NAFLD), liver cirrhosis, chronic viral hepatitis Β or C, HIV infection, inflammatory bowel diseases, irritable bowel syndrome and diverse autoimmune conditions [35]. Therefore, the maintenance of the integrity of the gut barrier is a crucial element for human health, and hence an increasing number of studies is conducted to understand the mechanisms involved in the improvement of the intestinal barrier integrity.

## 4. Non-Invasive Methods of Assessment of Gut Barrier in Clinical Trails

The important element in the gut barrier analysis is the selection of the appropriate analytical approach. Among various methods, the non-invasive techniques, which are based on the ingestion of specific molecules, or the analysis of circulating indirect markers which can evaluate the state of the gut barrier are the most commonly applied, especially in clinical trials. These methods can be easily applied in the clinical trials, in which the sampling of the tissues for histological analyses or local gene expression is impossible or too harmful. In this section, a brief presentation of the available methods is presented.

### 4.1. Absorption Methods for the Assessment of Gut Barrier Integrity

The integrity of the gut barrier can be assessed using various non-invasive approaches (Table 1). Among them, the most commonly used method in clinical trials is the sugar absorption test (SAT). SAT consists of the administration of a mixture of sugars with different molecular weights and different levels of penetration through the intestinal membrane. The most frequently used SAT includes the ingestion of high-molecular lactulose, which crosses the intestinal barrier to a small extent, through paracellular transport and low-molecular mannitol, which easily crosses the intestinal barrier by transcellular transport [36,37,38,39]. These molecules enter the bloodstream and then are excreted in the urine. The results of SAT are usually expressed as the ratio of lactulose to mannitol in the urine after 5–6 h of collection. The SAT accurately reflects the loss of intestinal barrier integrity in the small intestine [40]. The use of additional sugars, e.g., sucralose which is not metabolized by the gut microbiota, allows the assessment of integrity also in the colon [41]. Moreover, the ingestion of a certain amount of sucrose, which is easily metabolized by sucrases secreted by the duodenum, can be useful for the assessment of the integrity of the stomach and the proximal part of the duodenum. The sugars can be also replaced by other substances such as polyethylene glycols (PEG) with various molecular weights (400–4000 Da), ethylenediaminetetraacetic acid labelled with radioisotope chromium (^51^Cr-EDTA) and dextran labelled with fluorescein isothiocyanate (FITC-dextran) [40]. All these methods similar to SAT are based on the selective permeability of the gut barrier to molecules dependent on their size.

### 4.2. Methods for the Assessment of Gut Barrier Integrity Related to Bacterial Metabolism

Another group of methods used for gut barrier assessment is the analysis of the indirect markers being the products of bacterial metabolism. The products of bacterial metabolism used as an intestinal permeability marker are lipopolysaccharides (LPS), which are amphiphilic bacterial endotoxins that are a component of the outer cell membrane of gram-negative bacteria [42]. The presence of LPS reflects the increased gut permeability as a result of damage to the intestinal barrier. An alternative to the analysis of LPS concentration is the analysis of antibodies against the endotoxin core—EndoCAb (Endotoxin Core Antibodies) in the peripheral blood. This method allows the quantification of antibodies against the inner core of bacterial endotoxin, which is responsible for its toxicity. EndoCAb can be used as an indirect indicator of intestinal barrier damage since the penetration of endotoxin into the bloodstream is related to the dysfunction of intestinal mucosa integrity [43]. Finally, the last marker of bacterial metabolism is D-lactic acid. D-lactic acid is a product of the metabolism of many bacteria, including the host microbiota. Initially, D-lactate was considered an indicator of bacterial infections [44], but low levels of D-lactic acid are also found in healthy people. When the intestinal barrier is injured, the concentration of D-lactic acid in the bloodstream increases, which is related to its increased transit through the damaged intestinal mucosa. Thus, D-lactic acid can be used as an indicator of the impairment of the intestinal barrier. An increased concentration of D-lactic acid may also be associated with an increase in the number of bacteria in the gastrointestinal tract [45]. Therefore, the usefulness of this indicator for assessing intestinal permeability needs careful control.

### 4.3. Inflammation-Related Markers for the Assessment of Gut Barrier Integrity

The next group of intestinal permeability indicators are markers associated with inflammation. Zonulin is an analogue of the Zot toxin produced by *Vibrio cholerae*, which is involved in the regulation of TJ. Zonulin is an approximately 47 kDa protein that increases the permeability of the small intestine and contributes to the acquisition of primary intestinal immunity [42,46]. The molecular mechanism of zonulin action on the intestinal barrier is not fully understood. The Zot toxin activates the proteinase-activated receptor 2 (PAR2) followed by the epidermal growth factor receptor (EGFR) [47]. This signalling cascade results in the detachment of the peripheral ZO-1 protein from the occludins and claudins, the tight halo proteins [48]. It has been suggested that zonulin exhibits a similar mechanism of action, although this has not been conclusively confirmed. The concentration of zonulin in the bloodstream is elevated in the presence of disturbances in the integrity of the intestinal barrier, therefore zonulin is a useful marker of intestinal permeability [42,49].

The main enzyme catalysing the oxidation of diamines such as histamine, putrescine and cadaverine is diamine oxidase (DAO). This enzyme is synthesized in the intestinal mucosa, placenta, kidneys and thymus [50]. The majority of DAO in the bloodstream comes from the small intestine, where it is produced at the apical ends of the mature cells of the intestinal villi [51]. The determination of DAO activity was used to assess intestinal permeability in people suffering from Crohn’s disease, ulcerative colitis or acute lymphoblastic leukaemia [52].

Calprotectin is a 32-kDa acute-phase calcium and zinc-binding protein. It is produced by leukocytes, mainly by neutrophils, but also by macrophages and monocytes [53]. Inflammation overexpresses calprotectin in cells of the immune system. The concentration of this protein in serum and faeces increases, and under physiological conditions the concentration of calprotectin in the faeces exceeds that in the serum several times. Numerous studies have identified faecal calprotectin as a sensitive marker of intestinal inflammation [54,55,56]. Intestinal inflammation leads to an increase in intestinal permeability and the penetration of calprotectin-secreting leukocytes, which in turn leads to a significant increase in its concentration in the faeces. In practice, faecal calprotectin testing is used to distinguish IBD from Irritable Bowel Syndrome, to diagnose necrotising enteritis and monitor response to treatment of inflammatory bowel conditions [57].

Alpha-1-antitrypsin (AAT) is a 52 kDa trypsin inhibitor and is one of the most potent serine protease inhibitors. In the bloodstream, it is present at a concentration of 1.5 to 3.5 g/L, although, during acute inflammation, the concentration of AAT may increase many times [58]. This protein protects tissues against the influence of inflammatory cell enzymes, especially neutrophilic elastase [42]. In contrast with other serum proteins, AAT is highly resistant to gastrointestinal proteolytic enzymes and is excreted intact in the faeces. A high concentration of AAT is found in the stool due to inflammation and ulceration of the intestinal mucosa and increased intestinal permeability, as AAT passes from the bloodstream into the intestinal lumen. For this reason, AAT has been recognized as a marker of intestinal permeability [59]. AAT concentration can be determined using a nephelometer [60], although commercially available enzyme immunoassays are most commonly used for this purpose [42].

### 4.4. Markers Associated with the Damage of the Mucosa

Citrulline is a non-protein amino acid, a derivative of ornithine, which is converted into arginine in the urea cycle. The concentration of citrulline in the plasma under physiological conditions is 20–50 μmol/L. Citrulline present in the bloodstream is produced mainly by cells of the small intestine. Therefore, the concentration of this amino acid in the blood is considered an indicator of the activity of enterocytes (functional enterocyte mass, FEM). The reduction of FEM in course of many pathological conditions is connected with increased permeability of the gut barrier and leads to a decrease in the level of citrulline in the bloodstream. Numerous studies have shown a reduced level of citrulline in the plasma of people suffering from short bowel syndrome, HIV, adenovirus infections and during small intestine transplant rejection [61]. It should be emphasized that the proper functioning of the kidneys has a great influence on the concentration of citrulline in the bloodstream. Increased levels of citrulline have been found in people with moderate renal impairment [62], therefore the concentration of creatinine, which informs about the condition of the kidneys, should be monitored during the determination of citrulline.

Glutathione S-transferases (GSTs) are a family of detoxification enzymes that catalyse the nucleophilic coupling reactions of reduced glutathione with electrophilic compounds [63]. The catalytic activity of GSTs is associated with the detoxification of chemical compounds with electrophilic properties and reactive products of oxidative stress. Depending on the tissue they come from, there are four subgroups of GST: α, µ, π and θ. αGST is mainly present in the liver, kidneys and intestines. αGST has been proposed as a marker of intestinal epithelial cell damage [64]. Elevated levels of αGST in plasma and urine may indicate damage to the intestinal barrier, but also to the liver and kidneys since this enzyme is found in the epithelial cells of these organs. A disadvantage of αGST as a marker of intestinal barrier damage is its low specificity and it can be useful only if other diseases have been eliminated.

Fatty Acid Binding Proteins (FABPs) are small (14–15 kDa), water-soluble cytosolic proteins. Their function is to bind and transport fatty acids. There are several types of FABP, distinguished according to the tissues of origin [65]. Three types of fatty acid binding proteins have been found in the digestive tract: hepatic FABP (L-FABP) present in the liver, kidneys and intestines, bile acid binding proteins (BABPs) present in the ileum, and intestinal FABP (I-FABP) present mainly in the jejunum, and to a lesser extent in the colon. I-FABP is a 15-kDa protein produced in mature cells of the epithelium of the small intestine. Its function is to transfer fatty acids from the apical membrane of the enterocyte to the endoplasmic reticulum, where the biosynthesis of lipid complexes occurs. Due to its low molecular weight and relatively good solubility, I-FABP is easily released into the bloodstream when the intestinal epithelial membrane is damaged and is rapidly cleared by the kidneys (half-life is 11 min) [66]. Therefore, its presence can be measured in urine or plasma. The most common methods for determining I-FABP are enzyme immunoassay methods (ELISA). The physiological level of I-FABP reflects the exchange rate of intestinal epithelial cells [67], while an elevated level of I-FABP may indicate damage to the intestinal barrier [68]. Numerous studies have indicated that I-FABP can be considered a marker of epithelial cell damage. Increased levels of I-FABP in the urine have been found in people suffering from intestinal ischemia, systemic inflammatory response syndrome or necrotizing enterocolitis [67,69]. Due to the diversity of FABPs depending on the tissue from which they originate, the measurement of individual proteins in urine and/or plasma can be a valuable tool in locating tissue damage.

## 5. Principles of Polyphenols Action on the Gut Barrier

Dietary modification affects significantly the microbial ecosystem, which can influence the intestinal barrier [70,71]. For instance, it was reported that the Western diet aggravates the integrity of the gut barrier via modifications of the bacterial ecosystem, while individuals on the Mediterranean diet, rich in fruits, vegetables and unrefined cereals were found to have intact intestinal barriers [70]. The metabolites suggested to be involved in this process are short-chain fatty acids (SCFA), such as acetic, butyric, propionic and valeric acids, which are suggested as important factors in the functioning of the epithelium [72]. Recently, an increasing interest in the role of phenolic compounds, which are also consumed in large quantities in the Mediterranean diet, is observed in the gut barrier integrity [73,74].

The hypothesis about the involvement of phenolic compounds on the gut barrier results from the antioxidant and anti-inflammatory properties of these compounds. Since phenolic compounds are capable to downregulate inflammatory genes such as NF-κβ, reduce the production of cytokine production and promote the internal antioxidant capacity [75], it can be assumed that phenolic compounds can locally reduce inflammation. The NF-κβ signalling is strongly connected with cytokines (which activate the NF-κβ) and the gut barrier functions (by TJ impairment) [76]. Other signalling pathways, which are affected by phenolic compounds and can influence the gut barrier are various kinases, which are regulators of epithelial cells, and TJ expressions. Some phenolic compounds were found to inhibit different kinases involved in the phosphorylation of proteins involved in gut barrier functioning, such as protein kinase C (PKC) and myosin light-chain kinase (MLKC) [75,77].

Moreover, phenolic compounds were repeatedly reported to modulate the intestinal microbiota [76,78,79]. This two-way interaction not only facilitates the metabolism of phenolic compounds but also phenolic compounds can stimulate the growth of beneficial intestinal bacteria [78,79]. The derivatives originating from phenolic compounds microbial metabolism may not only affect backwards the bacteria but can also influence the signalling pathways [80]. Another potential way of action is the role of phenolic compounds in the metabolism of other molecules present in a colon, including SCFA, sterols and products of bacterial metabolism, which exhibit pro- or anti-inflammatory properties [81].

## 6. Effect of Resveratrol on the Gut Barrier

### 6.1. In Vitro Studies

The main evidence about the potential positive effects of resveratrol for the gut barrier is based on the in vitro studies, which were focused on the changes in the regulatory pathways. The most commonly used cell line was Caco-2 [82,83,84,85] and intestinal porcine enterocytes (IPEC-J2) [86,87]. The intestinal permeability in these studies was assessed by the measurement of transepithelial electrical resistance (TEER) across the cellular monolayer and by the measurement of the expression of the genes and corresponding proteins.

Carasco-Pozo et al. [82] reported that phenolic compounds applied in the Caco-2 cells with indomethacin-induced disruption of epithelial integrity were able to reverse the functioning of the layer. Among the analysed compounds, quercetin was the most efficient, followed by epigallocatechin gallate and resveratrol. Only rutin was found to be neutral on the cell integrity. In another study, resveratrol was applied in 1, 10 and 20 µM in Caco-2 cells treated with hydrogen peroxide to induce hyperpermeability and oxidative stress [83]. The authors found that resveratrol increased the epithelial expression and phosphorylation of ZO-1 and occludins with the increasing dose of polyphenol. Additionally, resveratrol protected Caco-2 cells from oxidative stress, reducing the accumulation of malonaldehyde and oxygen species and increasing the expression of enzymes involved in protection against oxidative stress, such as superoxide dismutase and heme oxygenase-1. 20 µM of resveratrol was able to block the hydrogen peroxide-induced activation of PKC and phosphorylation of p38, which had a confirmation in the protein expression assessed by Western blot.

Two studies showed that resveratrol is capable to protect the intestinal barrier from disruption caused by deoxynivaleron, a mycotoxin produced by *Fusarium* genera [86,87]. Both these studies showed that resveratrol prevents the deoxynivaleron-induced degradation of TJ proteins, and decreases TEER and bacteria translocation. The modulation of IL-6 and IL-8 secretion via mitogen-activated protein kinase-dependent pathways was proposed as a potential route of resveratrol activity.

A recent study evaluated the effect of resveratrol and its metabolites resulting from microbiota metabolism, namely dihydroresveratrol and 3-(4-hydroxyphenyl)-propionic acid on the intestinal barrier in LPS-treated Caco-2 cells [85]. It was found that only resveratrol and 3-(4-hydroxyphenyl)-propionic acid have beneficial effects on the intestinal barrier by increasing the expression of TJ protein and Muc2, and the molecular mechanism proposed by authors to be involved is monophosphate-activated protein kinase (AMPK)-mediated activation of CDX2 and the regulation of the SIRT1/NF-κβ pathway. At the same time, dihydroxyresveratrol did not show any positive effect on the intestinal barrier. Another study on the resveratrol derivative was conducted by Jo et al. [84]. The authors applied oxyresveratrol to the Caco-2 cells. Oxyresveratrol was found to upregulate the expression of genes and proteins related to TJ, such as claudin-1, ZO-1 and occludins. The improvement of the intestinal barrier by oxyresveratrol was associated with the activation of mitogen-activated protein kinase (MAPK) and PKC pathways.

### 6.2. Animal Studies

The results obtained in vitro needed confirmation in vivo, especially considering that resveratrol was applied directly on the cell lines, while in vivo the limited bioavailability of phenolic compounds has to be taken into consideration. The majority of studies were performed with rodents including Wistar and Sprague Dawley rats [83,88,89,90,91] and mice [85,92,93,94,95,96,97,98]. One study was performed with ducks [99]. Collectively, animal studies confirmed the beneficial effect of resveratrol on the gut barrier.

Wang et al. [83] reported that resveratrol restored intestinal integrity in rats with induced obstructive jaundice. The results of SAT in groups fed with resveratrol were significantly reduced compared to rats with obstructive jaundice and were closer to the control group. Moreover, the activity of superoxide oxidase was increasing with increasing doses of resveratrol (from 10 to 20 mg/kg) and in rats fed with 20 mg/kg was at the same level as in the control group. In another study, male Sprague-Dawley rats with high-fat diet (HFD)-induced NASH were fed with resveratrol for six weeks [91]. Resveratrol inhibited the development of NASH as well as attenuated the gut microbiota dysbiosis, colon inflammation and metabolic endotoxemia. An increase in the count of *Akkermansia muciniphila*, *Ruminococcaceae*, *Lachnospiraceae*, and a decrease in *Desulfovibrio* was noted in rats after resveratrol intake. Importantly, resveratrol ingestion led to the upregulation of TJ expression and decreased intestinal permeability, assessed based on bacterial translocation. HFD was used also to induce insulin resistance in mice [94]. The authors reported that resveratrol intake improved the lipid profile parameters in HFD-treated mice, as well as ameliorated endotoxemia, intestinal barrier dysfunction, glucose intolerance and inflammation. Similar to the previous study, the abundance of *Akkermansia* was increased after resveratrol administration. The confirmation of the positive effect of resveratrol on the gut barrier was found also in a study by Wang et al. [95], who observed the alleviation in HFD-induced NAFLD, improvement of intestinal barrier functions and gut microbiota composition after resveratrol intake. The expression of the TJ proteins and the intestinal mucosa morphology was improved after resveratrol administration, despite the HFD. The study conducted with cyclophosphamide-induced immunosuppressed mice confirmed that resveratrol can restore the intestinal microbiota dysbiosis, improve the gut barrier integrity and upregulate the expression of ZO-1, claudin-1 and occludin [98]. The authors confirmed that the TLR4-NF-κβ signalling pathway is involved in the resveratrol effect on the gut barrier. A very recent study by Hao et al. [97] evaluated the effect of resveratrol on intestinal permeability in mice exposed to aluminium chloride. Aluminium exposure induced depressive-like behaviour and increased intestinal permeability. The administration of resveratrol decreased depressive symptoms and upregulated the expression of SIRT1, which was found to reduce inflammation and restore intestinal permeability.

Finally, the study conducted with ducks with LPS-induced intestinal dysfunction showed that resveratrol in a dose of up to 500 mg/kg alleviated intestinal permeability established based on the increased expression of TJ genes and proteins [99]. The increasing dose of resveratrol resulted in the decrease of pro-inflammatory cytokine (IL-6, IL-18, TNF-α) levels and the expression of genes involved in inflammation (TLR4/NF-κβ signalling pathway, IKK, TXNIP, NLRP3, Caspase-1, IL-6, IL-18).

Interestingly, a recent study showed that resveratrol can act beneficially not only on the intestinal epithelium, but can also improve pulmonary epithelium functions. Alharris et al. [100] evaluated the effect of resveratrol in ovalbumin-induced murine allergic asthma. Resveratrol intake attenuated allergic asthma, improved pulmonary functions, and reduced inflammation in the lungs in ovalbumin-treated mice. Moreover, the administration of resveratrol affected not only the lung but also the intestinal tract. The administration of resveratrol led to changes in intestinal microbiota with the enrichment of *Bacteroides acidifaciens* and SCFA concentration in the colon.

The assumption about the positive effects of resveratrol on the gut barrier is mainly based on the gene and protein expression and TEER. More studies with functional tests of the circulating level of indirect metabolites (see Section 4) need to be performed to show the effects of the expression modifications.

### 6.3. Human Studies

To date, there is a limited number of studies on the effect of phenolic compounds on the gut barrier. Although some studies have been registered in clinical trial databases [74], the number of available published reports is very low. The MaPLE (Microbiome mAnipulation through Polyphenols for managing Leakiness in the Elderly) project is an example of a study focused on the interaction between polyphenols, microbiota and gut barrier [101]. The authors reported that 700 mg of total polyphenols daily in a form of three small portions of selected polyphenol-rich foods daily for 8 weeks reduced the intestinal permeability expressed as a circulating level of zonulin in the elderly population [102,103]. Notably, the analysis of intestinal permeability was based on only the level of zonulin, without any functional tests, such as SAT. To date, despite the successful findings obtained with in vitro and in vivo studies, no clinical studies were performed aimed to evaluate the effect of resveratrol on the gut barrier. However, considering the positive effects of resveratrol on inflammation [104,105,106], oxidative stress [105,107] and gut microbiota composition [104,108], and the involvement of these factors in the gut barrier integrity, it can be assumed that the positive effect of resveratrol on the intestinal permeability obtained with in vitro and in vivo studies may be observed also in clinical trials. In addition, the studies using functional tests, not only indirect markers are needed to fully address the integrity of the gut barrier.

## 7. Summary and Future Perspectives

To summarize, the current studies showed that phenolic compounds, including resveratrol, have a great potential to improve the integrity of the gut barrier. Very promising studies have been conducted with in vitro and animal models, establishing the expression of TJ genes and proteins or TEER. Still, more clinical trials must be performed to evaluate the effect of resveratrol on the gut barrier, especially in individuals with increased intestinal permeability, using both indirect markers and functional tests. Moreover, considering the two-way interaction of this phytochemical with microbiota and the important role of gut microorganisms in the functioning of the intestinal barrier, the interplay between all these three compartments should be carefully evaluated in future.

## Figures and Tables

**Table 1:** The characteristics of the methods for assessments of intestinal permeability.

Method	Analysed Marker	Localization	Type of Sample	Analytical Method	Disadvantages
Sugar absorption test (SAT)	Sugars of various molecular weights (lactulose, mannitol, sucralose, sucrose, raffinose)	Small intestine (can be extended to other parts of the intestinal tract by adding additional sugars to the mixture)	Urine	Chromatography	Time-consuming; requires chromatographical equipment for sugars analyses
PEG 400/4000	Polyethylene glycols (PEG)	The whole intestinal tract	Urine	Chromatography	Time-consuming; requires chromatographical equipment which can analyse PEG; big individual variation in response
^51^Cr-EDTA	Isotopically labelled EDTA	The whole intestinal tract	Urine	Chromatography	Radioactivity
FITC-dextran	Fluorescent-labelled dextran	The whole intestinal tract	Urine/serum	Chromatography/fluorimeter	Other substances (i.e., bilirubin) can give a fluorescence response
LAL	Endotoxin LPS	The whole intestinal tract	Plasma	ELISA	Low concentration—requires portal vein blood collection
EndoCAb	Antibodies anty-LPS	The whole intestinal tract	Serum	ELISA	Only in acute phase inflammations
D-lactate	Bacterial lactic acid	The whole intestinal tract	Plasma	ELISA/chromatography	Low specificity
Zonulin	Zonulin protein	-	Serum	ELISA	
DAO	Diamine oxidase	Small intestine	Plasma	ELISA	Requires the administration of heparin
Calprotectin	Calprotectin	Colon	Faeces	ELISA	Low specificity
AAT	Alfa-1-antitrypsin	Small intestine	Faeces/serum	ELISA	Unknown specificity
Citrulline	Citrulline	Small intestine	Plasma	ELISA/chromatography	Time-consuming; requires chromatographical equipment for amino acids analyses
GST	Glutathione S-transferases	-	Plasma/urine	ELISA	Low specificity
FABP	Fatty acid-binding proteins	Depending on the type of FABP	Plasma/urine	ELISA	Only in acute phase inflammations

## Data Availability

Not applicable.
